# Role of BDNF-mTORC1 Signaling Pathway in Female Depression

**DOI:** 10.1155/2021/6619515

**Published:** 2021-02-09

**Authors:** Xianquan An, Xiaoxiao Yao, Bingjin Li, Wei Yang, Ranji Cui, Guoqing Zhao, Yang Jin

**Affiliations:** ^1^Jilin Provincial Key Laboratory on Molecular and Chemical Genetic, Second Hospital of Jilin University, Changchun 130041, China; ^2^China-Japan Union Hospital of Jilin University, China

## Abstract

Depression is a common psychological and mental disorder, characterized by low mood, slow thinking and low will, and even suicidal tendencies in severe cases. It imposes a huge mental and economic burden on patients and their families, and its prevention and treatment have become an urgent public health problem. It is worth noting that there is a significant gender difference in the incidence of depression. Studies have shown that females are far more likely to suffer from depression than males, confirming a close relationship between estrogen and the onset of depression. Moreover, recent studies suggest that the brain-derived neurotrophic factor- (BDNF-) mammalian target of rapamycin complex-1 (mTORC1) signaling pathway is a crucial target pathway for improving depression and mediates the rapid antidepressant-like effects of various antidepressants. However, it is not clear whether the BDNF-mTORC1 signaling pathway mediates the regulation of female depression and how to regulate female depression. Hence, we focused on the modulation of estrogen-BDNF-mTORC1 signaling in depression and its possible mechanisms in recent years.

## 1. Introduction

Depression is a kind of mood disorder characterized by persistent depression, slow thinking, and decreased will activity. It is worth noting that the incidence of depression has significant gender differences. Because of the physical and social characteristics, the number of women suffering from depression worldwide is about twice that of men [1]. After puberty, females are more likely to suffer from depression than males, and the prevalence rate of females is significantly higher than men [2]. Other studies have shown that females exhibit depressive-like behaviors during periods of rapid estrogen decline, such as premenstrual, prenatal, postpartum, and perimenopausal periods [3–5]. Therefore, the function and regulation of estrogen are inevitably closely involved in the incidence of depression.

There is convincing scientific evidence that estrogen has neuroregulatory and neuroprotective effects, which are directly related to emotion. Studies have shown that estrogen levels in depressed females are lower than those in normal females, and persistently low levels of estrogen are closely related to the occurrence of depression. Estrogen can directly act on related brain regions and regulate the expression of target genes related to emotional and cognitive functions through classical nuclear receptor pathways. Preclinical studies have shown that bilaterally ovariectomized mice can be used as an effective estrogen deficiency-induced depression animal model and show a significant increase in depressive-like behaviors in the forced swimming test [6, 7]. 17*β*-Estradiol increased the expression of brain-derived neurotrophic factor (BDNF) in the prefrontal cortex (PFC), alleviated despair, and enhanced pleasure in ovariectomized female mice [8]. Depressive behaviors in females during the rapid decline of estrogen levels are closely related to the widespread distribution of estrogen receptors in emotionally related brain areas such as the hippocampus, PFC, and amygdala [9, 10]. At the same time, the antidepressant-like effects induced by 17-estradiol were absent in estrogen receptor *β* knockout mice but did not show significant changes in *α*-receptor knockout mice [11]. The increase of depression-like behavior in mice induced by estrogen deficiency was mainly related to the estrogen receptor. Clinically, the susceptibility to depression increases during the transitional period of menopause and early after the last menstruation [12]. Moreover, the quality of life of postmenopausal depression patients is significantly lower [13]. But evidence from clinical studies suggests that hormone treatment, especially estradiol, has successfully alleviated depression [14, 15]. Depressive symptoms in young men were also involved with elevated estradiol levels [16]. These data further support the view that estrogen levels are critical in the pathobiology of affective disorders.

## 2. BDNF-mTORC1 Signaling Pathway

BDNF is a member of neurotrophic factors, a family of proteins that are essential for the growth and survival of neurons. It is playing an increasingly pivotal part in the pathophysiology of depression and the therapeutic mechanism of related antidepressants. Preclinical studies have shown that bilateral ovariectomy as an effective depression model induced by estrogen deficiency significantly decreased BDNF levels in the hippocampus and PFC [9, 10]. This suggested that increased depressive-like behaviors in mice induced by estrogen deficiency are primarily related to ER*β*. Further studies found that the BDNF level in the brain of estrogen receptor *β* knockout mice was remarkably reduced, while that in the brain of estrogen receptor *α* knockout mice was little changed [17]. Therefore, the estrogen receptor *β*-BDNF signaling pathway may mediate the regulation of depressive-like behaviors in female mice.

mTORC1 is a major growth regulator, whose signal pathway is closely related to synaptic plasticity; that is, it affects dendrites and dendritic spines by controlling the synthesis of proteins related to synaptic formation [18]. Hence, the mTORC1 signaling pathway is closely related to the synaptic structure and function plasticity. Researchers found that inhibition of the mTOR signal delayed the onset of puberty in female rats to some extent [19]. Moreover, expression decrease of mTORC1 and its upstream or downstream proteins, as well as inhibition of 1synaptic growth and regulation, in brain regions such as the hypothalamus, PFC, and hippocampus of ovariectomized murine, was reversed by estrogen administration [20–22]. These mean that the mTOR signaling pathway is indeed related to the regulation of estrogen, particularly in the central nervous system (CNS). Beyond that, downregulation of the mTORC1 pathway and synaptic changes have also been found in a variety of other models of depression in murine [23–25]. Likewise, clinical studies have also found decreased levels of mTORC1 expression and decreased synaptic formation in the PFC of depressed patients [26]. All of these indicate that the antidepressant effects mediated by the mTORC1 signaling pathway may also be closely related to the classical neural circuit.

Although there is no evidence to suggest a specific mechanism by which estrogen regulates mTORC1, BDNF is a key regulator. Recent researches have led to discoveries that the considerable upstream pathways of mTOR in the brain are PI3K/Akt/mTORC1 [27], MEK/ERK/mTORC1 [28], and LKB1/AMPK/mTORC1 [29]. As the upstream of LKB1 activation, the role of extracellular BDNF is realized by the upregulation of intracellular cAMP [30]. Meanwhile, chronic restraint stress reduced levels of mTORC1 and its downstream effectors such as 4E-BP-1 and p70S6K in the rat hippocampus, which is antagonized by antidepressants, escitalopram and paroxetine [31].

BDNF has been shown to affect the nervous system through the BDNF-mTORC1 pathway. In several reports, ketamine and scopolamine enhance the number and maturity of synapses by activating the BDNF-mTOR pathway to upregulate the expression of various synapse-related proteins, while blocking mTOR signals can completely interrupt the occurrence and behavioral response of these synapses [32–35]. It may be a unique fast-acting antidepressant mechanism. Studies have also manifested that hypidone hydrochloride activates pyramidal neurons by relieving the inhibitory effect of 5-HT_1A_ receptors on GABAergic neurons and then acts on the BDNF-mTORC1 pathway to exert an antidepressant role [36, 37]. These findings make mTORC1 an attractive therapeutic target for depression. For example, NV-5138, a novel antidepressant (a mTORC1 activator), enhances mTORC1 signaling and increased the number, function, and protein levels of synapses in the PFC, in which BDNF is required to participate [38]. In turn, the fast-acting antidepressant effects of ketamine and its active metabolite (2R,6R)-hydroxyketamine were blocked by BDNF function-blocking antibody or rapamycin [39, 40], a classical inhibitor of the mTORC1 [41], suggesting that researches on mTORC1 will help in the further development of antidepressants.

## 3. Role of BDNF-mTOR1 Signaling Pathway in Depression

### 3.1. BDNF-mTORC1 Signaling Pathway and Rapid Antidepressant Effects

Briefly, the potential mechanism of rapid antidepressant action of the BDNF-mTORC1 signaling pathway may be as follows: first, glutaminergic neurons release glutamate by inhibiting the activity of GABA interneurons; then, the AMPA receptor and VDCC were further activated to promote the release of BDNF; finally, the release of BDNF activates TrkB, Akt, ERK, AMPK, etc., and then activates the mTORC1 pathway, which promotes increases in proteins involved in synaptic formation (e.g., GluA1 and PSD95) and further increases the frequency and amplitude of the excitatory postsynaptic current (EPSC), thus promoting the growth of neurons and synapses to play an antidepressant-like role [34, 42, 43]. Although the mTORC1 pathway is considered to be an effective therapy for depression at present, there is still a separate report in which mTORC2, but not mTORC1, is required for hippocampal mGluR-LTD and associated behaviors [44], and further research is needed to investigate the role of mTORC2.

### 3.2. BDNF-mTORC1 Signaling Pathway and Autophagy

Autophagy is a conservative process of maintaining cellular energy and protein homeostasis [45]. It can effectively eliminate damaged proteins and organelles associated with certain diseases [46], but overactivated autophagy can also damage cells. Therefore, whether autophagy plays a positive or negative role in regulating neurological diseases is still a matter of debate [47]. What is certain is that autophagy dysfunction may lead to a variety of neurological disorders, such as depression, epilepsy, and Alzheimer's disease [48–50]. mTORC1 is a key molecule in autophagy, which can inhibit autophagy by competitively occupying ULK1 [51]. Its activated pathways such as Akt and MAPK signaling pathways inhibit autophagy, while negatively regulated pathways such as AMPK and P53 signaling pathways promote autophagy [52–54]. This indicates that mTOR is a key regulatory component in the relationship between depression and autophagy. And the mTOR signaling pathway indeed exerts neuroprotective effects by regulating autophagy and inducing nerve regeneration by promoting protein synthesis [55].

According to studies, autophagy regulates depression bidirectionally. On the one hand, obvious excessive autophagy activation during some depression results in the decline of the survival rate of neurons and glial cells and neuronal apoptosis [56]. Some antidepressants effectively function by improving this activated autophagy through the mTOR pathway [57]. Patchouli alcohol can inhibit excessive autophagy, repair synapses, and restore hippocampal autophagy flux by activating the mTOR signaling pathway, thus preventing depressant-like behaviors induced by CUMS [58]. Interestingly, BDNF promotes neuron survival by activating mTOR signaling to improve excess autophagy flux [59]. Besides, the BDNF-TrkB pathway also participates in the regulation of autophagy. For instance, the BDNF-TrkB pathway regulates antidepressant-like actions of H2S and fluoxetine by enhancing hippocampal autophagy [49, 60]. The neuroprotection of BDNF in vitro is also performed by inhibiting autophagy through the PI3K-Akt-mTOR pathway [53]. The regulation of autophagy by local BDNF-mTOR may also affect synaptic plasticity since the suppression of mTOR in stimulated neurons causes AMPA receptor degradation in spines through autophagy [52].

On the other hand, it has also been proved that autophagy between neurons is impaired in depression, which can be alleviated by pharmacologic enhancement of autophagy [61]. The majority of antidepressants may kick in through the upregulation of autophagy [62]. Ketamine, a quick-acting antidepressant, is an example [63], although its enhancement of mTOR activity has been confirmed. As one of the most abundant and bioactive constituents in vitamin E, *α*-tocopherol showed antidepressant-like effects on mice through the upregulation of autophagy mediated by the mTOR-AMPK pathway [54]. Trehalose may work on depression due to its ability to enhance autophagy as well [64]. Therefore, the BDNF-mTORC1 pathway can indeed regulate depression through autophagy, but its specific mechanism remains to be studied.

### 3.3. BDNF-mTORC1 Signaling Pathway and Monoamine Neurotransmitters

According to the monoaminergic hypothesis, lack of monoamine neurotransmitters such as 5-HT, DA, and NE in the brain may cause depression [65]. Estrogen deficiency has been shown to have significant effects on monoaminergic systems, including 5-HT, DA, and NE [66]. As an instance, the anxiety-like behavior caused by food restriction may be mediated by the decreased activation of estrogen receptor *β* in the serotonergic dorsal raphe nucleus neurons, which may be due to the decrease of the estrogen level [67]. It has been found that the disturbance of estrogen balance during menopause results in the imbalance of the BDNF-5-HT_2A_ signal and the decrease of synaptic plasticity, which puts the brain in a depressed state [17]. Furthermore, 17*β*-estradiol preferentially acts as an antidepressant by regulating levels of multiple neurotransmitters, dopaminergic receptors, serotonergic receptors, and the sigma-1 receptors expressed in the CNS to regulate neurotransmitter systems [68–70]. Thus, the monoaminergic system exerts the vital regulatory part in female depression.

Studies have shown that the rapid activation of the mTOR pathway is a significant medium for the rapid antidepressant action of ketamine and scopolamine [34]. Other studies have revealed that selective stimulation of the 5-HT_1A_ receptor in the medial PFC has also been shown to alleviate depressant-like behaviors [71, 72]. This may be through the activation of the AMPA receptor-BDNF-mTOR signal, thereby enhancing the synaptic function of mPFC [73]. In another study, scopolamine can increase the concentration of 5-HT and dopamine neurotransmitter system in the brain and cause delirium symptoms, while selective 5-HT_1A_ antagonist reverses it to some extent through the induction of PI3K-Akt-mTORC1 [74]. Besides, the inhibition of rapamycin on the Akt-mTOR pathway blocked the change of 5-HT_2AR_ signal transduction mode [75]. 20(S)-Protopanaxadiol and liquiritigenin may also have antidepressant effects through normalization of monoamine neurotransmitter and corticosterone (CORT) levels and enhancement of the BDNF-mTOR pathway [76, 77]. The interaction between the 5-HT_6_ receptor and mTOR pathway was also found; that is, 5-HT_6_ receptor activation can increase mTOR signal in rodent PFC. In connection with cognitive impairment, rapamycin, an mTOR inhibitor, can reverse the increase of mTOR activity in PFC like a 5-HT_6_ antagonist, thus improving cognitive disorder induced by 5-HT_6_ agonists [78]. All these demonstrated the interaction between the BDNF-mTORC1 pathway and the monoaminergic system in the occurrence and treatment of depression.

### 3.4. BDNF-mTORC1 Signaling Pathway and Neuroendocrine System

Hyperactivity and stress feedback disorder of the HPA axis is particularly considerable in the pathogenesis of depression, which may be improved by regulating the homeostasis of the HPA axis. On the one hand, there is a close relationship between the activities of the hypothalamic-pituitary-adrenal (HPA) axis and the hypothalamic-pituitary-gonad (HPG) axis, and they interact in estrogen-mediated affective disorders. CNS regulates the synthesis and secretion of estrogen through the HPG axis, while estrogen regulates the functions of the pituitary and hypothalamus through the HPA axis in a feedback way, thus affecting the levels of stress hormones like corticotropin- (ACTH-) releasing hormone, ACTH and CORT [79], and thereby relieving the emotional stress of postmenopausal women [80].

**Figure 1:**
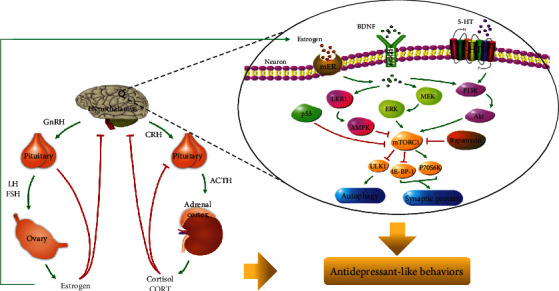
The regulatory effects of the estrogen-BDNF-mTORC1 signaling pathway in depression. Green arrows indicate activation; T-shaped red arrows indicate inhibition. Abbreviations: 5-HTR: 5-hydroxytryptamine receptor; ACTH: corticotropin; AMPK: AMP-activated protein kinase; Akt: serine/threonine protein kinase; BDNF: brain-derived neurotrophic factor; CRH: corticotropin-releasing hormone; ERK: extracellular signal-regulated kinase; GnRH: gonadotropin-releasing hormone; LH: luteinizing hormone; LKB1: liver kinase B1; MEK: mitogen-activated extracellular signal-regulated kinase; mER: membrane estrogen receptor; mTORC1: mammalian target of rapamycin complex-1; PI3K: PI-3 kinase; TrkB: tyrosine kinase B.

On the other hand, studies have confirmed that neurotrophins such as BDNF are involved in neuroendocrine regulation [81]. An early study in adult rats found that continuous BDNF administration into the ventricle affected activity and biological rhythm of the HPA axis [82]. In a later study, knockdown of BDNF by siRNA in rats inhibited the expression of endogenous BDNF in different brain areas as well as weakened the growing level of ACTH and CORT caused by normal stress [83]. A recent study found that patients with two separate BDNF single nucleotide polymorphism alleles (rs2049046 and rs11030094), beneficial alleles associated with antidepressant responses, had significantly lower cortisol responses to dexamethasone suppression/CRH tests at discharge [84]. These prove the vital function of BDNF in regulating the HPA axis. Furthermore, some drugs exert antidepressant-like and neuroprotective effects in this way. The improvement of Apelin-13 in chronic stress depressive-like behaviors was achieved through upregulation of BDNF by improving the HPA axis and hippocampal glucocorticoid receptor disorder [85]. The reduction of depressive-like behavior in mice treated with CSDS can be alleviated by dammarane saponin through the restoration of monoamine neurotransmitter levels and HPA axis, which is achieved in part by increasing the BDNF-mTOR pathway [86]. Similarly, water extract of Vaccinium bracteatum leaf showed neuroprotective effects by increasing phosphorylation of CREB in CORT-induced cell damage mediated by the mTOR signaling pathway [87]. Cortisol induces PC12 cell injury by blocking autophagy mediated by the AMPK-mTOR pathway [88, 89]. Autophagy activated AMPK activator metformin and mTOR inhibitor rapamycin, and chlorogenic acid significantly reduced CORT-induced PC12 cytotoxicity by activating autophagy [88, 89]. The potential regulatory role of the estrogen-BDNF-mTORC1 signaling pathway in depression is shown in Figure 1.

## 4. Conclusion

Overall, there are more and more innovative researches on the pathogenesis of depression, which offers hope for the quality of life for patients. The BDNF-mTORC1 signaling pathway is considered to be an important target pathway for rapid antidepressant therapy, which plays a beneficial role in female depression. Next, further search for drugs acting on the BDNF-mTORC1 pathway or allosteric modulators of mTORC1 is of great significance to improve its role in the pathology of depression, which will greatly improve the situation of female patients.

## Data Availability

Data are available upon request.
